# An Unusual Case of Obturator Hernia Detected in an Elderly Man by Computed Tomography

**DOI:** 10.7759/cureus.8775

**Published:** 2020-06-22

**Authors:** Pham Hong Duc, Nguyen Thi Thai Hoa, Dang Quang Hung, Huynh Quang Huy

**Affiliations:** 1 Radiology, Hanoi Medical University, Hanoi, VNM; 2 Internal Medicine, Vietnam National Cancer Hospital, Hanoi, VNM; 3 Radiology, Saint-Paul Hospital, Hanoi, VNM; 4 Radiology, Pham Ngoc Thach University of Medicine, Ho Chi Minh City, VNM

**Keywords:** obturator hernias, computed tomography, intestinal obstruction

## Abstract

Obturator hernia is a rare condition, characterized by the herniation of an intestinal segment between the obturator and the pectineus muscles through the obturator foramen. Obturator hernias usually occur in the elderly and are less common in males than in females, with a male-to-female ratio of about 1/14. In recent years, the use of diagnostic imaging, especially CT, to determine the causes of intestinal obstruction has been improved to allow for an early and accurate diagnosis, even of obturator hernias, which are extremely rare in male patients. We report a thin elderly man, without a history of surgery and with chronic constipation and an unremarkable Howship-Romberg sign, which was correctly diagnosed before surgery as an obturator hernia using CT.

## Introduction

Obturator hernia is a rare condition that accounts for approximately 1%-3.9% of all abdominal hernias and 0.4%-1.6% of all cases of mechanical intestinal obstruction [1-5]. In addition, the incidence of obturator hernia is higher in Asia than in the West [3]. Obturator hernia occurs more often in older women and is referred to as the ‘little old lady’s hernia’ [3,6-8]. Hernia contents are usually in the small intestine, mainly ileum, in 71%-95% of cases, and the common morphology is a Richter-type hernia, which is found in 56%-61% of cases [2-5]. It occurs more frequently on the right side, as the sigmoid colon overlies the obturator foramen on the left side, with a right/left ratio of 1.3-5:1 [3-5,7,8]. Obturator hernias have been described as bilateral hernias in about 6% of cases [3].

In the literature, obturator hernia is most often reported in men. Clinical diagnosis is usually based on patients with predisposing factors, but in men presenting an obturator, the hernia is rarely posed, which may lead to delays in diagnosis, possibly causing fatal complications in patients. Herein, we report an unusual case of an elderly man with a small body, chronic constipation, and unremarkable clinical symptoms of an obturator hernia, according to preoperative diagnosis using CT.

## Case presentation

A 76-year-old thin and frail man (BMI = 18.1 kg/m^2^), who visited the emergency department with complaints of an acute onset of right-side groin pain, followed by diffuse abdominal distension with pain, lasting for one week. He also complains of nausea, mild vomiting, and constipation for the past several days. No other significant medical history was reported. On examination, his abdomen distended with tenderness on palpation of the right lower abdomen, with no peritoneal signs. The digital rectal examination showed a normal stool. A local examination was not noted as an inguinal hernia or fullness in the right groin region and a Howship-Romberg sign. The laboratory findings were unremarkable, with mild leukocytosis (11 G/L).

**Figure 1:**
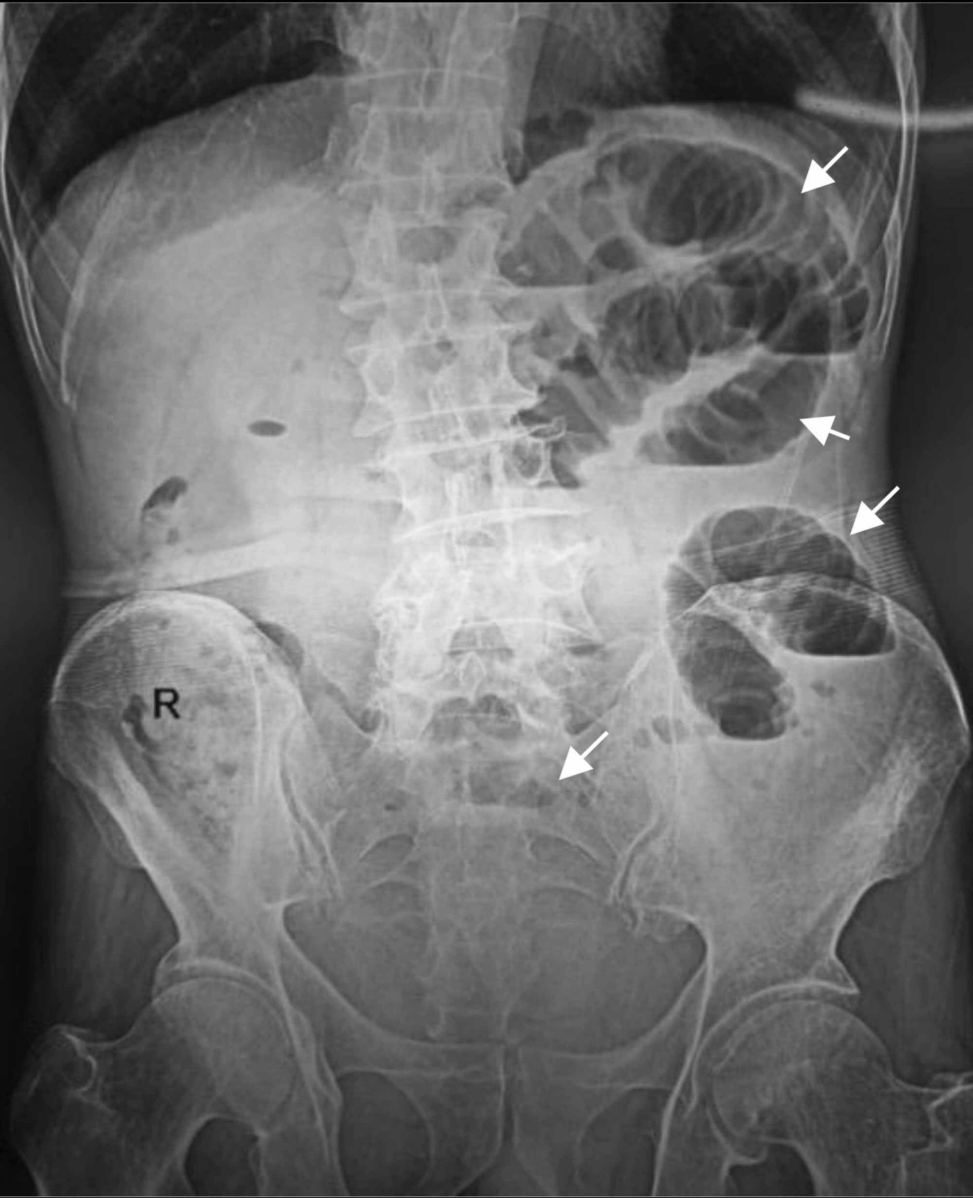
Flat abdominal X-ray X-ray of the abdomen standing, recording multiple air-fluid levels of dilated small bowel loops on the left abdominal side

**Figure 2:**
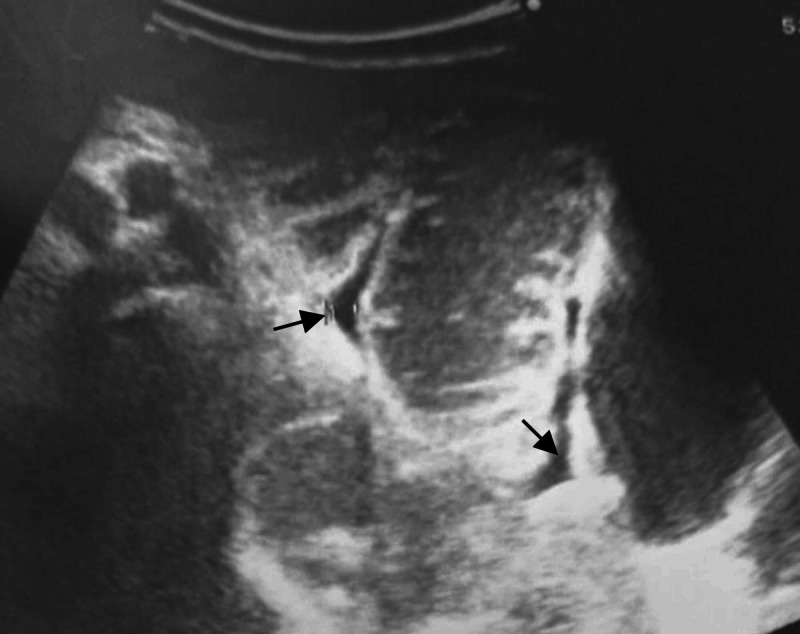
Abdominal ultrasound Ultrasound showed the dilated small bowel loops with a little fluid between the intestinal loops

**Figure 3:**
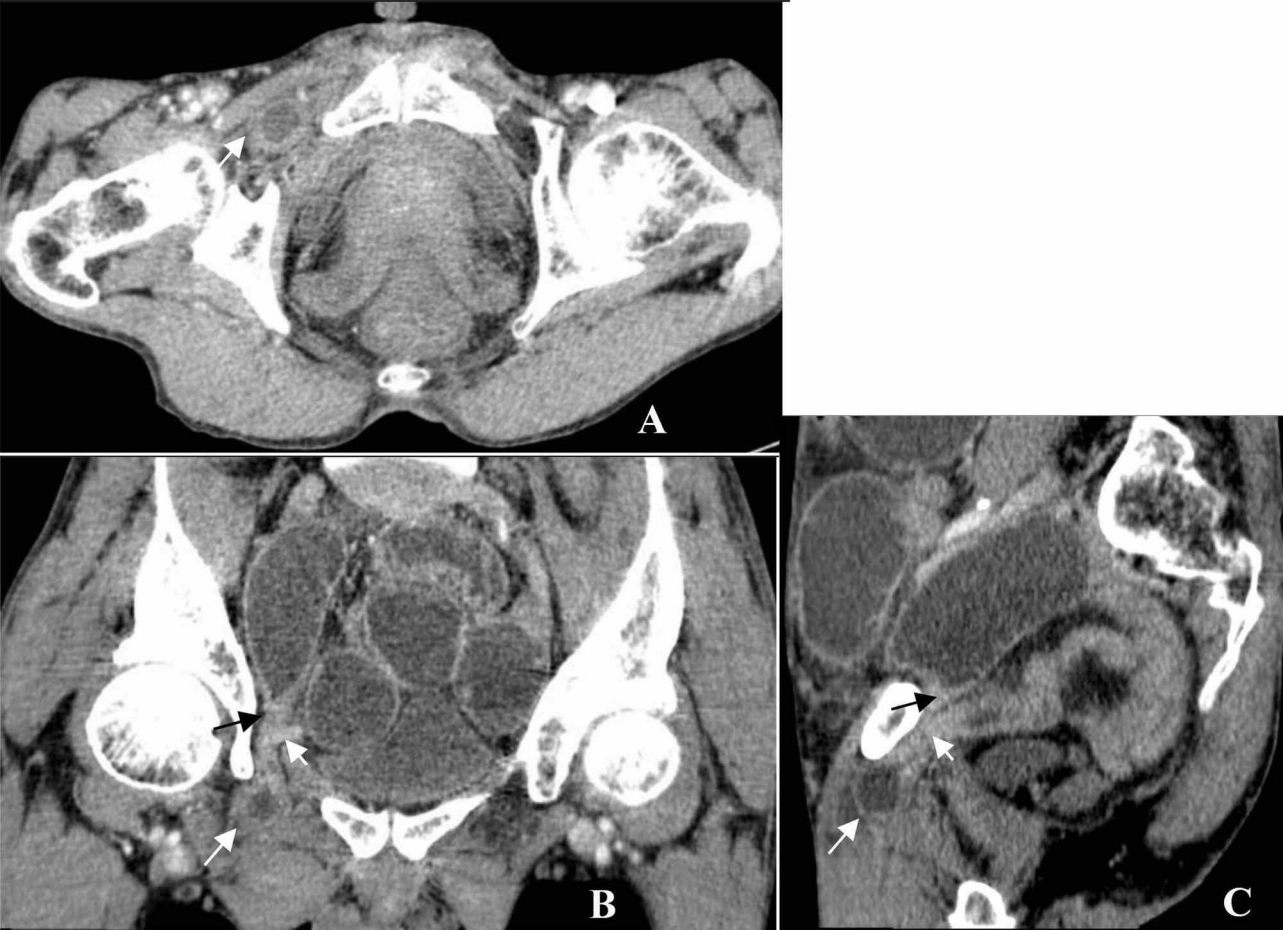
Abdominal contrast-enhanced computed tomography Abdominal contrast-enhanced CT with venous phase in axial (A), coronal (B), and sagittal (C) reconstruction, showing small bowel loops with a thick and non-significant enhanced wall entering the right obturator foramen (long arrow). The inner obturator canal shows a beak sign of the dilated proximal loops (black arrow) and the collapsed distal loops (short arrow)

Initial abdominal radiographs showed evidence of dilated small bowel loops with multiple air-fluid levels, consistent with an obstruction with no pneumoperitoneum (Figure 1). An ultrasound of the abdomen showed markedly dilated small bowel loops with increased peristalsis, and a small amount of free fluid was noted in between the small bowel loops, but the cause of the obstruction could not be identified (Figure 2). The patient was introduced for an emergency CT scan of the abdomen and pelvis. Contrast-enhanced computed tomography (CECT) revealed that the cause of the intestinal obstructions was the ileal loop entering through the right obturator foramen and lying between the pectineus muscle anteriorly and external obturator muscle posteriorly. The wall of this incarcerated loop hernia also showed thickening and less enhancement than the dilated loops in the abdomen (Figure 3).

**Figure 4:**
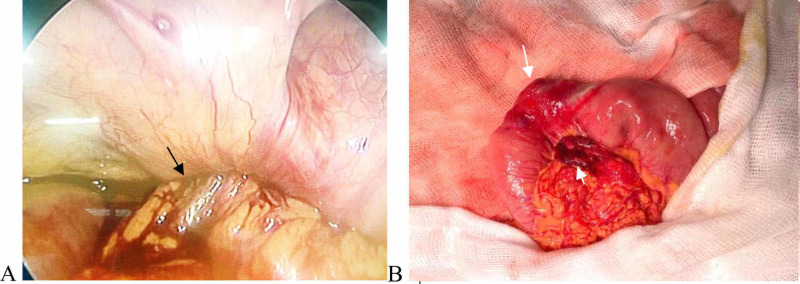
Laparoscopic operation Laparoscopy (A) revealed the ileal segment entering the right obturator canal (black arrow). Laparotomy (B) presented the incarcerated loop hernia, showing pre-gangrenous changes (long arrow), including necrosis of its mesentery (short arrow)

A diagnosis of the right-side obstructed obturator hernia was made. The patient was optimized and admitted to emergency laparoscopic surgery. Laparoscopy confirmed the left obstructed obturator hernia, and forceps could not be used to pull it into the abdominal cavity. Then, laparotomy via a midline incision was carried out, showing that the incarcerated intestinal hernia as a Richter-type hernia protruding through the right obturator canal was an ileal loop of 3-4 cm and located 60 cm from the ileocecal junction. There were pre-gangrenous changes, including necrosis of its mesentery and a constriction ring in that bowel segment, which was resected, followed by anastomosis (Figure 4). The patient had an unstable postoperative period and was discharged on postoperative day seven, without complications.

## Discussion

In summarizing the articles that we considered in our literature review, we found that favorable factors predisposed patients to hernia formation, including a larger obturator foramen in the wider pelvis, a loss of the peritoneum fat stroma covering the obturator canal and a history of multiple births in an emaciated body, with concomitant medical illness and without previous abdominal surgery. Other factors associated with increased abdominal pressure include ascites, chronic constipation, and chronic obstructive pulmonary disease.

Diagnosis of an obturator hernia before surgery is difficult due to its non-specific symptoms and because there is usually intestinal obstruction. Clinical diagnosis based on the Howship-Romberg sign presented pain spreading along the medial thigh to the knee because of a nervous blockage by the hernia mass and pain increasing when the leg was spread out and rotated outside in 13%-68% of cases [1-5,8,9]. This sign may be confused as arthritis in the elderly [8]. Other Hannington-Kiff signs consisted of loss of reflexes of adductor muscles. However, the absence of these specific signs is not rare and may be found in up to 66%-87% of cases [3,8], with a suspected preoperative diagnosis on the basis of the clinical setting at only 38% [3], but associated with CT scan; this is increased to 100% [6,7].

The most prominent clinical condition of our patient was intestinal obstruction. While he had a thin body and frequent constipation and had not had previous abdominal surgery, we did not consider the possibility that it might be an obturator hernia at the hospital, because he was a man. Thus, the Howship-Romberg sign, as well as the Hannington-Kiff sign, were not recorded before the CT scan. These specific signs were also unclear at the clinical examination, after the established diagnosis of obturator hernia by CT scan [4]. Thanapaisan reported only 13% of patients presenting the Howship-Romberg sign, and the author proposed that this low incidence is possibly related to their being an incomplete history and physical examination, poor communication, and previous leg pain [4].

An abdominal X-ray and ultrasound only show the usual signs of bowel obstruction. An ultrasound can give more information if Valsalva's maneuver comparison is performed on the unaffected side of the subject, suspecting an abdominal wall hernia, but this seems to be only detected in cases of inguinal region hernias [10]. CT imaging is recognized as having an important role in finding the cause of intestinal obstruction in the elderly for preoperative diagnosis, with an accuracy of 87%-100% [1,2,5-7,9]. Technically, an axial CT image down to the upper thigh region should be taken. Obturator herniation on the CT findings may also be overlooked or misdiagnosed as a hernia, rather than a femoral hernia because this is the more common type of hernia in older women [11].

Abdominal and pelvic CECT present dilated bowel loops and fluid-air levels with transition points in the obturator foramen. Confirmation of an obturator hernia, when the CT findings were showing bowel herniation through the obturator foramen and lying between the pectineus muscle anteriorly and external obturator muscle posteriorly, may present an air-fluid level. The venous phase of CECT also provided additional information on the thickening and reduced enhancement of the intestinal wall, which could help to predict the gangrenous degree of the strangulated obturator hernia, as in our case.

The use of abdominal and pelvic CT associated with ultrasonography may increase the preoperative diagnostic accuracy and be particularly important in those patients with a negative Howship-Romberg sign [2]. The CT scan should be performed first in thin, elderly, and highly fertile women in cases of small bowel obstruction, and other examinations may be too hard and invasive and less accurate, including herniorrhaphy, ultrasonography, and gastrointestinal contrast medium [5,7,9]. Regardless of the presence or absence of the Howship-Romberg sign, early diagnosis with CT scans and subsequent surgery also produced good results [7,12]. Kammori et al. reported a retrospective study of a comparison of two groups who received and did not receive a CT scan, showing that the preoperative diagnostic accuracy of the group who received a CT scan was significantly higher than that of the other group. The authors reported that the use of CT scanning aided in early diagnosis and surgical intervention, improved patient outcomes and decreased the rate of gut resection, thus allowing for the proper treatment of this rare condition [2].

The incidence of multiple hernias is not rare, including indirect or indirect inguinal, femoral, and obturator hernias, accounting for 23% of cases [13]. Therefore, in a preoperative hernia diagnosis, it should be carefully considered that other combined hernias may be hidden by a larger one, avoiding omissions that increase the risk of fatal complications associated with emergency hernia surgery in the elderly [14].

## Conclusions

Obturator hernia is an uncommon condition, which is especially rare in men, and this fact may delay preoperative diagnosis, causing a high risk of late bowel complications. Therefore, in the case of a thin older male patient, without a history of surgery and with favorable factors, such as chronic constipation, it is necessary to think about finding specific signs, such as the Howship-Romberg sign, and to perform early abdomen and pelvic CECT scans that cover the upper thigh area, which would allow for an exact diagnosis and prediction of necrosis of the intestinal segment in strangulated obturator hernias.
